# Dr. Wm. Grosvenor Ring

**Published:** 1917-07

**Authors:** 


**Dr. Wm. Grosvenor Ring,** Buffalo 1884, died at his home in Buffalo, April 16, aged 57. He was the son of the late Dr. Wm. Ring of the class of 1847 and brother of the late Dr. Charles A. Ring, of the class of 1878. He was born in Buffalo, Nov. 10, 1860, and practiced there for nearly a third of a century. Death was quite sudden, following an acute exacerbation of a chronic valvular endocarditis. He leaves a widow and a son 6 years old.

